# Recurrent Hurthle cell thyroid carcinoma does not preclude long-term survival: a case report and review of the literature

**DOI:** 10.1186/s13256-021-02987-z

**Published:** 2021-08-11

**Authors:** Richard David Blossey, Dennis Kleine-Döpke, Kristina Imeen Ringe, Daniel Pöhnert, Bastian Ringe, Jürgen Klempnauer, Oliver Beetz

**Affiliations:** 1 Department of General, Visceral and Transplant Surgery, Hannover Medical School, Carl-Neuberg-Strasse 1, 30625 Hannover, Lower Saxony Germany; 2 Department of Diagnostic and Interventional Radiology, Hannover Medical School, Hannover, Germany

**Keywords:** Hurthle cell carcinoma, Follicular thyroid carcinoma, Multisite metastases, Unusual metastases, Pancreatic metastasis, Cardiac metastasis, Case report

## Abstract

**Background:**

Follicular thyroid carcinoma is the second most common malignancy of the thyroid gland. In 2016, the so-called Hurthle cell thyroid carcinoma, formerly known as the oxyphilic variant of the follicular thyroid carcinoma, was reclassified by the World Health Organization as a separate pathological entity, which accounts for approximately 3% of all thyroid cancers. Although Hurthle cell thyroid carcinomas are known for their more aggressive tumor biology, metastases are observed in a minority of cases, and long-term survival can be expected. However, disseminated disease is often associated with poor outcome.

**Case presentation:**

In the presented case, a 63-year-old Caucasian female was incidentally diagnosed with Hurthle cell thyroid carcinoma after undergoing hemithyroidectomy for a nodular goiter. Following completion thyroidectomy, two courses of radioactive iodine therapy were administered. After 4 years of uneventful follow-up, the patient gradually developed metastases in five different organs, with the majority representing unusual sites, such as heart, kidney, and pancreas over a course of 14 years. The lesions were either treated with radioactive iodine therapy or removed surgically, depending on iodine avidity.

**Conclusion:**

Follicular and Hurthle cell thyroid carcinoma are known to potentially spread hematogenously to typical sites, such as lung or bones, however; unusual metastatic sites as presented in our case can also be observed. A search of the literature revealed only scattered reports on patients with multiple metastases in unusual locations. Furthermore, the observed long-term survival of our patient is contradictory to the existing data. As demonstrated, recurrent disease may appear years after the initial diagnosis, emphasizing the importance of consistent aftercare. Radioactive iodine therapy, extracorporeal radiation therapy, and surgical metastasectomy are central therapeutic components. In summary, our case exemplifies that thorough aftercare and aggressive treatment enables long-term survival even in recurrent Hurthle cell thyroid carcinoma displaying unusual multisite metastases.

## Introduction

Thyroid carcinomas are responsible for approximately 1–2% of all malignant tumors. They are divided into differentiated (DTC) and nondifferentiated or anaplastic carcinomas. In addition, medullary thyroid carcinoma originates from calcitonin-producing cells [1].

DTC include papillary (PTC) and follicular (FTC) thyroid cancers. Together, these two entities account for 80–90% of all primary thyroid malignomas [2] and are associated with a favorable prognosis and relatively low mortality rates, respectively. This feature is primarily attributed to effective targeted therapy regimes: a combination of radioactive iodine (RAI) therapy and surgery [3]. Hurthle cell carcinomas (HCTC) were originally defined as oxyphilic or oncocytic variant of FTC, and account for 3% of all thyroid carcinomas [4]. In 2016, however, new findings led to a reclassification by the World Health Organization as a separate pathological entity [5, 6].

DTC differ not only in their histopathological structure, but also in their metastatic pathways. The majority of PTC metastasizes within the locoregional cervical and mediastinal lymphatic compartments, whereas patients suffering from FTC or HCTC primarily display hematogenous dissemination [7–9]. It is assumed that FTC and HCTC are based on an adenoma-carcinoma sequence [5]. The distinction between benign adenoma and malignant carcinoma is based on the histopathological occurrence of capsular or vascular invasion. There are different subtypes of FTC or HCTC, such as the minimally or the broadly invasive type, with the latter being associated with a worse prognosis [10, 11]. In addition to the invasion status, a distinction is also made by the grade of differentiation.

In general, FTC is known to metastasize less often than PTC. Metastatic lesions are mainly observed within the lungs, the bones, and the brain [12, 13]. HCTC shows similar metastatic patterns, although some authors report a trend to more frequent distant lesions [14]. To the best of our knowledge, there are only scattered reports of patients suffering from HCTC and multiple unusual site metastases. Hence, data on therapeutic strategies and (long-term) prognosis are lacking.

## Case report

**Fig. 1:**
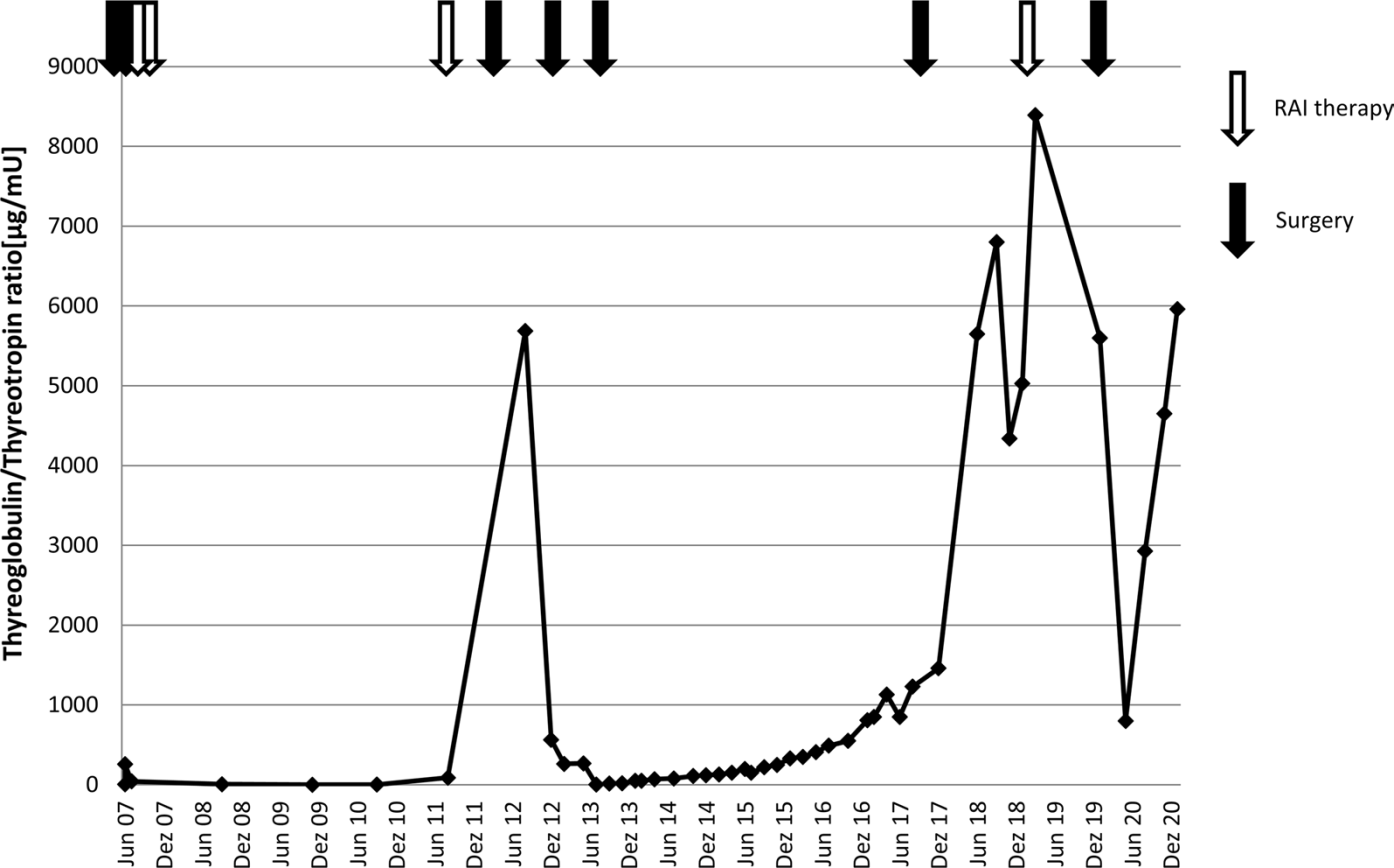
Ratio of thyroglobulin (TG) to thyrotropin (TSH) [µg/mU] at selected points in time during follow-up. Black arrows mark the surgical interventions. White arrows mark the radioactive iodine (RAI) therapies

**Fig. 2:**
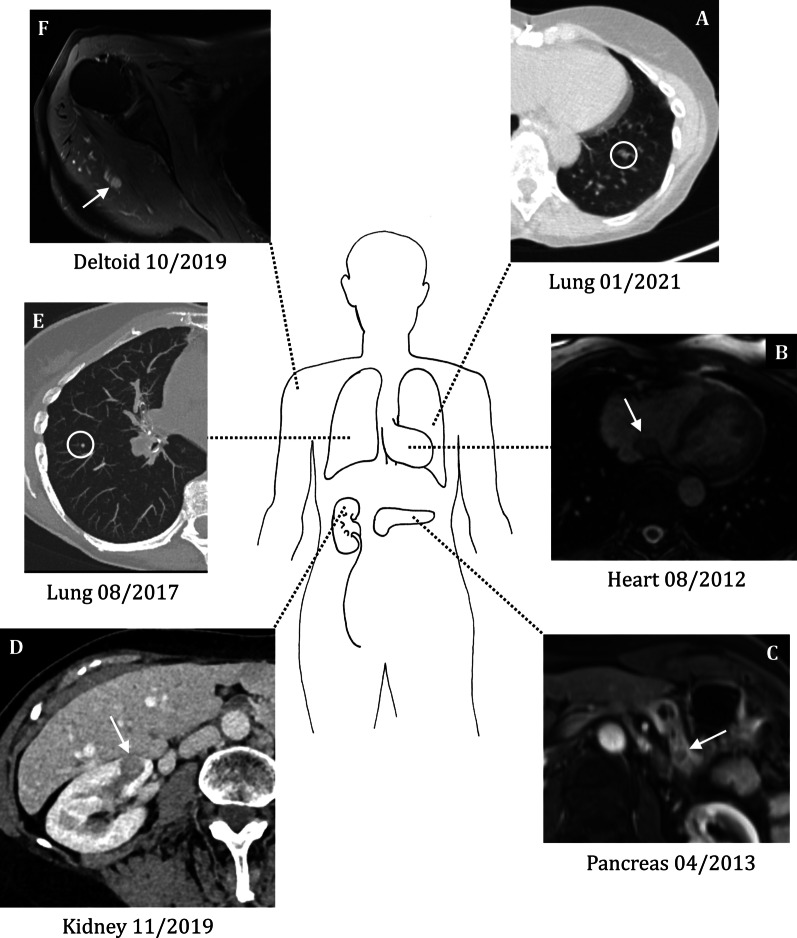
Schematic presentation of the metastatic site. **A** Axial computed tomography (CT) image of the thorax with the lung metastasis in the left lower lobe (white circle). **B** Axial magnetic resonance imaging (MRI) image of the thorax. The arrow marks the heart metastasis in the right atrium. **C** Axial MRI image of the upper abdomen depicting the pancreatic tail metastasis (arrow). **D** Axial CT image of the abdomen with metastasis in the right superior renal pole (arrow). **E** Axial CT image of the chest. The white circle marks one of the metastases in the right upper lobe. **F** Axial MRI image of the right shoulder, depicting a contrast enhancing the metastasis in the deltoid muscle (arrow)

We report the case of a 63-year-old Caucasian female patient who was incidentally diagnosed with an oxyphilic variant of a minimally invasive FTC (syn. Hurthle cell carcinoma; HCTC) after undergoing left hemithyroidectomy with subtotal resection of the contralateral side for a nodular goiter in January of 2007. In line with current guidelines, thyroidectomy of the right side was completed and lymphadenectomy of the central cervical lymphatic compartment was performed in February 2007. The initial tumor formula was pT2, pN0 (0/6), cM0 with an oxyphilic phenotype. Subsequently, the patient received radioactive iodine (RAI) therapy (March 2007: 4.8 GBq; July 2007: 7.4 GBq). Unstimulated serum thyroglobulin (TG) concentration before the second course of RAI therapy was 2.2 µg/L (shown in Fig. 1). The follow-up period of 4 years did not reveal clinical symptoms or other signs of tumor relapse. In June 2011, we observed an increase in TG concentration of 13.0 µg/L (Fig. 1). Positron emission tomography–computed tomography (PET-CT) detected a mediastinal mass with increased tracer uptake, which was further confirmed by magnetic resonance imaging (MRI) revealing enlarged thoracic lymph nodes. Accordingly, RAI therapy was repeated in August 2011 (7.0 GBq). Shortly afterwards, stimulated TG concentrations increased again to 43.9 µg/L (Fig. 1). Further PET-CT showed focal tracer uptake between esophagus and thoracic aorta, which was interpreted as lymphatic metastases. Hence, regional lymphadenectomy and pericardiectomy were performed in January 2012 via lateral thoracotomy. Of the eight lymph nodes removed, none was positive for the known HCTC, and the TG concentration persistently increased. In August 2012, unstimulated TG was 170.0 µg/L (Fig. 1). Eventually, we decided to perform a cardiac MRI, revealing a pediculated mass located within the right atrial septum (shown in Fig. 2). In November 2012, a postoperatively confirmed HCTC metastasis was surgically removed from the right atrial septum via pericardial patch plastic. Only 6 months after the procedure, the unstimulated TG concentration was still increased at 48.3 µg/L (Fig. 1). PET-MRI showed a further metastasis located within the pancreatic body (Fig. 2). Thus, we performed a distal pancreatectomy in June 2013. Afterwards, another uneventful follow-up period of 3 years occurred. In August 2017, PET-MRI was performed because of a newly increasing unstimulated serum TG concentration, revealing metastases within the right lung and right deltoid muscle (Fig. 2).

In October 2017, the pulmonary metastases were removed via an open surgical wedge resection. Because of its small size, the muscular metastasis was initially left untreated.

In 2018, follow-up was unremarkable except for the known metastasis of the deltoid muscle, which remained stable in size. Unstimulated TG concentrations were elevated but stable. A year later, in May 2019, another PET-CT was carried out because of an increasing unstimulated TG concentration (112.0 µg/L) (Fig. 1). The scan revealed a new metastasis of the right upper pulmonary lobe apart from the metastasis within the deltoid muscle. In addition, we detected a rapidly progressive metastasis located in the upper pole of the right kidney (Fig. 2). RAI therapy (7.4 GBq) was performed. The pulmonary metastasis showed a good response, and the renal metastasis could not be visualized on postinterventional scintigraphy, but still observable on CT and PET-CT. After an interdisciplinary discussion, surgical removal of the upper pole of the right kidney and the no longer iodine-avid lesion of the deltoid muscle was performed in January 2020. In both locations, the pathological findings confirmed metastases of the known HCTC.

Now, almost 1 year after the last surgery, the patient is in good general condition and her quality of life is excellent. Unfortunately, unstimulated TG increased over the last few months (Fig. 1). A PET-CT scan revealed two new small pulmonary metastases in the right upper and left lower lobe (Fig. 2). Together we decided on a “wait-and-see” strategy. Follow-up imaging is planned in 3 months.

## Discussion and conclusion

In this case, we present the history of a patient suffering from an oxyphilic variant of a minimally invasive FTC, now known as HCTC. HCTC was long considered as an aggressive variant or subtype of FTC. In 2016, the World Health Organization reclassified HCTC as a separate pathological entity, although histological distinction of FTC and HCTC is particularly difficult [6]. Clinically, the HCTC is regarded as more aggressive and lethal than FTC, especially in older research articles. However, more recent work contradicts these assumptions. Nagar *et al*. were able to demonstrate that HCTC and FTC have a similar mortality [4]. It should be mentioned that the strict separation between FTC, PTC, and HCTC has only established itself internationally over the last 5–10 years. In the work of Cipriani *et al*., the histopathologic results of more than 60 FTC-cases from 1965 to 2007 were reviewed retrospectively by three pathologists [15]. The majority of the original classifications had to be corrected. Similar findings were reported by a Japanese workgroup after reviewing more than 440 FTC cases. Only 45% of the originally as FTC classified thyroid cancers were confirmed as such on the basis of current diagnostic criteria and genetic and histological techniques [16]. In summary, much of the early work on both FTC and HCTC has to be interpreted with caution since a reliable discrimination of both entities seems uncertain [17]. In our patient, we observed metastases in different organs and soft tissue (that is, lung, heart, kidney, pancreas, and skeletal muscle) over a period of more than 14 years. Retrospective analyses have reported that 1–3% of all DTC display distant disease [13, 18]. Although reports of patients experiencing more than three metastatic sites are scarce, Hugen *et al*. reported that over 60% of all patients with metastasized DTC have multiple metastases, corroborating our findings [19]. However, this was an autopsy study, and some of the included patients were not diagnosed with a thyroid malignancy during their lifetime, limiting the applicability of these results. In most cases, multiple metastases can be found within the lung and the bones [20]. Distant metastases and especially the involvement of multiple organs are risk factors for increased mortality [12, 21]. Although we also observed recurrent pulmonary metastases, the majority of the lesions represented rather unusual metastatic sites such as the pancreas, the heart, or the kidney [22–24]. A South Korean study investigated 38,772 patients with DTC, which were further divided into three groups: no metastases (38,361 patients; 97.94%), usual site metastases (392 patients; 1.01%), and unusual site metastases (19 patients; 0.05%). Contrary to our report, only six patients of the latter group experienced lesions in more than one unusual metastatic site. Furthermore, most of these patients were diagnosed with PTC, and the median survival (68 months) was markedly inferior to the observed survival of our patient (more than 168 months) [13]. A meta-analysis by Paspala *et al*., which included 11 patients with pancreatic metastases from thyroid carcinoma revealed an even worse median overall survival of only 37.6 months as opposed to the data presented in our report [22]. Stojadinovic *et al*. reported on a cohort of 260 patients with metastasized DTC in which one-third of all patients displayed multisite metastases [25], confirming data reported by Yoon *et al*. [13]. Prognostic factors for favorable outcome were female gender, radiological detection of metastases without clinical symptoms, a long disease-free interval between primary diagnosis and metachronous metastases, and young age. Apart from the latter, all of these factors were observed in our patient, partially explaining the excellent long-term survival. In a Japanese study of 106 individuals with metastasized FTC, disease-specific survival after 15 years was only 23.9%. Patient age was reported to be the most significant predictive factor for survival [26]. In contrast, despite the advanced age of our patient, she was able to withstand her disease for more than 14 years.

Equally important to the mentioned prognostic factors is a thorough and lifelong follow-up (including physical examination, ultrasound, radioiodine scintigraphy and cross-sectional imaging including ^18^F-FDG PET) and the combination of RAI therapy and radical surgery, in non-iodine-avid metastases. In patients not eligible for surgery, less invasive approaches such as microwave ablation or extracorporeal radiation and even multiple kinase inhibitors such as lenvatinib or sorafenib are effective alternatives [18, 27–29].

Furthermore, the presented case serves as an example of a structured interdisciplinary concept: Over the years, six different departments (radiology, nuclear medicine, cardiothoracic surgery, urology, trauma and orthopedic surgery, and visceral and endocrine surgery) participated in diagnostics and treatment. Experienced endocrine surgeons together with nuclear medicine practitioners should preside over such concepts as they are most familiar with the course of disease, possible changes in tumor biology and behavior, and surgical and conservative treatment modalities. A vital part in their cooperation is a lively discussion. Over the past few years, tumor boards became a strong and valid tool in tumor therapy. They improved the optimization of therapeutic decision-making and helped us to conquer different cancers, including thyroid malignomas, more sufficiently. Our report shows that recurrent disease in patients with HCTC does not preclude long-term survival if modern concepts of tumor aftercare and therapy are ensured.

The strength of this case report lies in the long follow-up of over 14 years and the durability in the treatment team. The patient was continuously cared for by the same experienced endocrine surgeon and nuclear practitioner. Furthermore, the patient showed a high level of compliance and adhered to the prescribed aftercare plans as well as the treatment suggestions. The limitation of this case report typically lies in the exclusive consideration of only one case. Additionally, the diagnostic uncertainty between the different thyroid carcinoma entities is an important fact.

## Data Availability

The datasets used in this case report are available from the corresponding author on reasonable request.

## Abbreviations

DTC: Differentiated thyroid carcinoma
FTC: Follicular thyroid carcinoma
HCTC: Hurthle cell thyroid carcinoma
RAI: Radioactive iodine
TG: Thyroglobulin
CT: Computed tomography
MRI: Magnet resonance imaging
PET: Positron emission tomography
^18^F-FDG: Fluorodeoxyglucose (^18^F)

## References

[1] Ferlay J, Soerjomataram I, Dikshit R, Eser S, Mathers C, Rebelo M,* et al*. Cancer incidence and mortality worldwide: sources, methods and major patterns in GLOBOCAN 2012. Int J Cancer. 2015;136(5):E359–86. 10.1002/ijc.29210 25220842

[2] Kilfoy BA, Zheng T, Holford TR, Han X, Ward MH, Sjodin A,* et al*. International patterns and trends in thyroid cancer incidence, 1973–2002. Cancer Causes Control CCC. 2009;20(5):525–31. 10.1007/s10552-008-9260-4 PMC2788231 19016336

[3] Schlumberger MJ. Papillary and follicular thyroid carcinoma. N Engl J Med. 1998;338(5):297–306. 10.1056/NEJM199801293380506 9445411

[4] Nagar S, Aschebrook-Kilfoy B, Kaplan EL, Angelos P, Grogan RH. Hurthle cell carcinoma: an update on survival over the last 35 years. Surgery. 2013;154(6):1263–71; discussion 71. 10.1016/j.surg.2013.06.029 23973114

[5] Daniels GH. Follicular thyroid carcinoma: a perspective. Thyroid. 2018;28(10):1229–42. 10.1089/thy.2018.0306 30039751

[6] Lloyd RVOR, Klöppel G, Rosai J. WHO classification of tumours of endocrine organs. 4^th^ edn. International Agency for Research on Cancer; 2017.

[7] Cabanillas ME, McFadden DG, Durante C. Thyroid cancer. Lancet (London, England). 2016;388(10061):2783–95. 10.1016/S0140-6736(16)30172-6 27240885

[8] Shaha AR, Ferlito A, Rinaldo A. Distant metastases from thyroid and parathyroid cancer. ORL. 2001;63(4):243–9. 10.1159/000055749 11408821

[9] Shaha AR, Shah JP, Loree TR. Patterns of nodal and distant metastasis based on histologic varieties in differentiated carcinoma of the thyroid. Am J Surg. 1996;172(6):692–4. 10.1016/s0002-9610(96)00310-8 8988680

[10] Grani G, Lamartina L, Durante C, Filetti S, Cooper DS. Follicular thyroid cancer and Hürthle cell carcinoma: challenges in diagnosis, treatment, and clinical management. Lancet Diabetes Endocrinol. 2018;6(6):500–14. 10.1016/S2213-8587(17)30325-X 29102432

[11] Machens A, Holzhausen HJ, Dralle H. The prognostic value of primary tumor size in papillary and follicular thyroid carcinoma. Cancer. 2005;103(11):2269–73. 10.1002/cncr.21055 15856429

[12] Ruegemer JJ, Hay ID, Bergstralh EJ, Ryan JJ, Offord KP, Gorman CA. Distant metastases in differentiated thyroid carcinoma: a multivariate analysis of prognostic variables. J Clin Endocrinol Metab. 1988;67(3):501–8. 10.1210/jcem-67-3-501 3410936

[13] Yoon JH, Jeon MJ, Kim M, Hong AR, Kim HK, Shin DY,* et al*. Unusual metastases from differentiated thyroid cancers: a multicenter study in Korea. PLoS ONE. 2020;15(8): e0238207. 10.1371/journal.pone.0238207 PMC7447021 32841270

[14] Besic N, Schwarzbartl-Pevec A, Vidergar-Kralj B, Crnic T, Gazic B, Marolt MM. Treatment and outcome of 32 patients with distant metastases of Hürthle cell thyroid carcinoma: a single-institution experience. BMC Cancer. 2016;16:162. 10.1186/s12885-016-2179-3 PMC4768323 26921186

[15] Cipriani NA, Nagar S, Kaplan SP, White MG, Antic T, Sadow PM,* et al*. Follicular thyroid carcinoma: how have histologic diagnoses changed in the last half-century and what are the prognostic implications? Thyroid. 2015;25(11):1209–16. 10.1089/thy.2015.0297 PMC4948203 26440366

[16] Hirokawa M, Ito Y, Kuma S, Takamura Y, Miya A, Kobayashi K,* et al*. Nodal metastasis in well-differentiated follicular carcinoma of the thyroid: its incidence and clinical significance. Oncol Lett. 2010;1(5):873–6. 10.3892/ol_00000154 PMC3436349 22966397

[17] Bhattacharyya N. Survival and prognosis in Hürthle cell carcinoma of the thyroid gland. Arch Otolaryngol Head Neck Surg. 2003;129(2):207–10. 10.1001/archotol.129.2.207 12578450

[18] Farina E, Monari F, Tallini G, Repaci A, Mazzarotto R, Giunchi F,* et al*. Unusual thyroid carcinoma metastases: a case series and literature review. Endocr Pathol. 2016;27(1):55–64. 10.1007/s12022-015-9410-7 26662609

[19] Hugen N, Sloot YJE, Netea-Maier RT, Van de Water C, Smit JWA, Nagtegaal ID,* et al*. Divergent metastatic patterns between subtypes of thyroid carcinoma results from the nationwide Dutch pathology registry. J Clin Endocrinol Metab. 2020;105(3):e299-306. 10.1210/clinem/dgz078 PMC7112975 31641763

[20] Mazzaferri EL, Massoll N. Management of papillary and follicular (differentiated) thyroid cancer: new paradigms using recombinant human thyrotropin. Endocr Relat Cancer. 2002;9(4):227–47. 10.1677/erc.0.0090227 12542401

[21] Elisei R, Molinaro E, Agate L, Bottici V, Masserini L, Ceccarelli C,* et al*. Are the clinical and pathological features of differentiated thyroid carcinoma really changed over the last 35 years? Study on 4187 patients from a single Italian institution to answer this question. J Clin Endocrinol Metab. 2010;95(4):1516–27. 10.1210/jc.2009-1536 20156922

[22] Paspala A, Kostakis ID, Gaitanidis A, Prodromidou A, Schizas D, Machairas N. Long-term outcomes after hepatic and pancreatic resections for metastases from thyroid cancer: a systematic review of the literature. J Gastrointest Cancer. 2019;50(1):9–15. 10.1007/s12029-018-00196-4 30618003

[23] Catford SR, Lee KT, Pace MD, Marasco SF, Longano A, Topliss DJ. Cardiac metastasis from thyroid carcinoma. Thyroid. 2011;21(8):855–66. 10.1089/thy.2010.0273 21751883

[24] Liou MJ, Lin JD, Chung MH, Liau CT, Hsueh C. Renal metastasis from papillary thyroid microcarcinoma. Acta Otolaryngol. 2005;125(4):438–42. 10.1080/00016480410022822 15823819

[25] Stojadinovic A, Shoup M, Ghossein RA, Nissan A, Brennan MF, Shah JP,* et al*. The role of operations for distantly metastatic well-differentiated thyroid carcinoma. Surgery. 2002;131(6):636–43. 10.1067/msy.2002.124732 12075176

[26] Sugino K, Kameyama K, Nagahama M, Kitagawa W, Shibuya H, Ohkuwa K,* et al*. Follicular thyroid carcinoma with distant metastasis: outcome and prognostic factor. Endocr J. 2014;61(3):273–9. 10.1507/endocrj.ej13-0437 24420337

[27] Wertenbroek MW, Links TP, Prins TR, Plukker JT, van der Jagt EJ, de Jong KP. Radiofrequency ablation of hepatic metastases from thyroid carcinoma. Thyroid. 2008;18(10):1105–10. 10.1089/thy.2008.0080 18816179

[28] Segkos K, Schmidt C, Nabhan F. Isolated liver metastasis in Hürthle cell thyroid cancer treated with microwave ablation. Case Rep Endocrinol. 2017;2017:2790741. 10.1155/2017/2790741 PMC5253501 28163939

[29] Lee GM, You JY, Kim HY, Chai YJ, Kim HK, Dionigi G,* et al*. Successful radiofrequency ablation strategies for benign thyroid nodules. Endocrine. 2019;64(2):316–21. 10.1007/s12020-018-1829-4 30569260

